# MAO-B inhibition by selegiline blunts cardiac functions improved by high-fat diet: Role of inflammation, apoptosis, and calcium-handling

**DOI:** 10.1016/j.crphar.2025.100237

**Published:** 2025-11-11

**Authors:** Szabolcs Hambalkó, Csilla Pelyhe, Csenger Kovácsházi, Bence Kenyeres, Bernadett Kiss, Bence Ágg, Tamás G. Gergely, Bennet Y. Weber, András Makkos, Gábor B. Brenner, Tímea Komlódi, László Tretter, Csaba Horváth, Izabela Jarabicová, Adriana Adameová, Anikó Görbe, Paola Poggi, Alexandros Chatgilialoglu, Ildikó Horváth, Domokos Máthé, Krisztián Szigeti, Attila Oláh, Tamás Radovits, Zoltán V. Varga, Béla Merkely, Gary F. Baxter, Rainer Schulz, Péter Ferdinandy, Zoltán Giricz

**Affiliations:** a Semmelweis University, Department of Pharmacology and Pharmacotherapy, 1085, Budapest, Hungary; b MTA-SE System Pharmacology Research Group, Department of Pharmacology and Pharmacotherapy, Semmelweis University, 1089, Budapest, Hungary; c Heart and Vascular Center, Semmelweis University, Budapest, Hungary; d HCEMM-SU Cardiometabolic Immunology Research Group, Semmelweis University, 1089, Budapest, Hungary; e Department of Biochemistry, Semmelweis University, 37-47 Tuzolto Street, Budapest, 1094, Hungary; f Department of Pharmacology and Toxicology, Faculty of Pharmacy, Comenius University, Odbojárov 10, 832 32, Bratislava, Slovak Republic; g Centre of Experimental Medicine, Institute for Heart Research, Slovak Academy of Sciences, Bratislava, Slovak Republic; h Pharmahungary Group, 6722, Szeged, Hungary; i Remembrane S.r.L., Imola, Italy; j School of Pharmacy and Pharmaceutical Sciences, Cardiff University, Redwood Building, King Edward VII Avenue, Cardiff, CF10 3NB, UK; k Institute of Physiology, Justus-Liebig University Giessen, Giessen, Germany; l Department of Biophysics and Radiation Biology, Semmelweis University, Budapest, Hungary; m In Vivo Imaging Advanced Core Facility, Hungarian Center of Excellence for Molecular Medicine (HCEMM), 1094, Budapest, Hungary; n HUN-REN Physical Virology Research Group, Semmelweis University, Tűzoltó utca 37-47, 1094, Budapest, Hungary

**Keywords:** Heart, Obesity, Obesity paradox, Selegiline, SERCA2a, Calcium homeostasis

## Abstract

**Background:**

Obesity is a major risk factor for the development of cardiovascular disease. However, recent research shows that moderate obesity reduces the risk of developing cardiovascular disease. We evidenced before that MAO-B inhibitor selegiline reduced visceral adiposity.

**Aim:**

Therefore, our aim was to investigate cardiac effects of selegiline in moderate obesity in rats treated with a high-fat diet (HFD).

**Key findings:**

We demonstrated that HFD improved cardiac contractility parameters, which were reversed by selegiline. Enhanced contractility might be attributed to an increased sarcoplasmic/endoplasmic reticulum Ca^2+^-ATPase (SERCA2a) expression and phospholamban pentamerization. Selegiline reduced SERCA2a expression in HFD. HFD increased Tumor necrosis factor and Nuclear factor-kappa B expression which were not affected by selegiline. HFD induced proapoptotic processes, which were restored by selegiline.

**Conclusion:**

In conclusion, moderate obesity improves cardiac function through Ca^2+^ homeostasis and inflammatory processes and MAO-B inhibition reverses these effects.

## Key messages

• Moderate obesity improves cardiac contractile functions in HFD-fed rats.
• Both moderate obesity and selegiline modulates Ca^2+^ homeostasis.
• Moderate obesity enhances some markers of inflammation in the heart.
• Moderate obesity increased apoptosis, which was attenuated by selegiline.
• Selegiline has an adverse effect on the obesity paradox observed in moderate obesity.

## Graphical abstract

**Figure:**
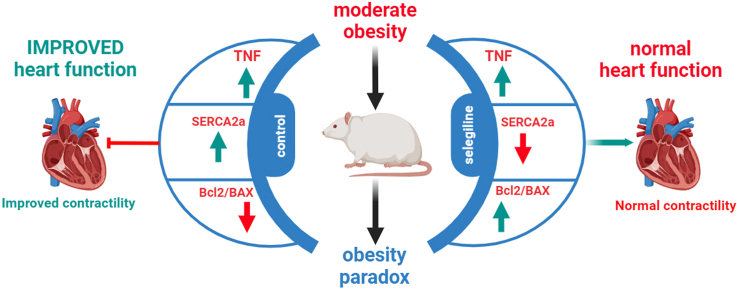

## 1 Introduction

Metabolic disorders, such as obesity and hyperlipidemia are associated with low-grade chronic inflammation and increased oxidative stress, which are the two major mechanisms underlying cardiometabolic diseases (CMD) (McMurray et al., 2016a; Pereira and Alvarez-Leite, 2014; Yang et al., 2006, 2008). While obesity remains a major risk factor for cardiovascular disease, several epidemiological studies have demonstrated a positive correlation between moderate obesity and cardiac function and improved outcomes of cardiovascular diseases (Antonopoulos and Tousoulis, 2017). This phenomenon is referred to as the obesity paradox. The term obesity paradox has been defined in numerous publications as a clinical phenomenon wherein individuals with overweight or obesity diagnosed with pre-existing cardiovascular disease exhibit better prognoses than non-obese patients. In addition, certain metabolically healthy patients with obesity are less prone to developing cardiovascular disease than patients with severe obesity or even lean individuals. Although the seemingly paradoxical effect of obesity on cardiac health has been well described in clinical scenarios, investigations in animal models are scarce, and its molecular mechanism remains unclear. In light of this knowledge gap, we examined the major cardiac molecular pathways implicated in obesity.

Under physiological conditions, reactive oxygen species (ROS) production regulates signaling pathways associated with cytoprotection, clearance of abnormal mitochondria as well as mitochondrial fission (Zorov et al., 2014). However, excessive amounts of ROS damage mitochondria, and other organelles (Schieber and Chandel, 2014; Montgomery and Turner, 2015) and promote apoptosis (de Mello et al., 2018). Furthermore, excessive ROS can lead to oxidative damage to ion channels, including sarcoplasmic/endoplasmic reticulum calcium ATPase 2a (SERCA2a), which may result in disturbances in cardiac Ca^2+^ homeostasis (Balderas-Villalobos et al., 2013). Therefore, targeting ROS production may further help to alleviate CMDs.

Monoamine oxidases (MAOs) are enzymes involved in the degradation of monoamines, which affects various physiological processes (Bortolato et al., 2008). MAOs produce reactive oxygen species (ROS) as a by-product, making them a major source of oxidative stress (Kaludercic et al., 2011). MAO-B inhibition with selegiline has been shown to reduce oxidative stress and improve various conditions, including obesity (Nagy et al., 2018) and cardiac disease (Qin et al., 2003; Takahata et al., 2006). However, the effects on energy balance and ROS production or whether it may interfere with the beneficial effects of the obesity paradox in the heart are still unclear.

Adequate energy metabolism and Ca^2+^ homeostasis are is crucial for cardiac contractile function. Hyperlipidemia and obesity have been shown to deteriorate Ca^2+^ homeostasis (Hamilton and Terentyev, 2018) through altered expression and phosphorylation of a key protein of the Ca^2+^ cycling, SERCA2a, and its main regulator phospholamban (PLN) (Lai et al., 2022). However, there is a lack of information in the literature on the effect of moderate obesity or obesity paradox on Ca^2+^ homeostasis. Since MAO-B inhibition could preserve mitochondrial health, it might further help to restore impaired Ca^2+^ homeostasis in hyperlipidemia.

Therefore, the aim of this study was to investigate the effect of moderate obesity on cardiac function, cell death- and survival mechanisms, and Ca^2+^ homeostasis. In addition, we investigated whether MAO-B inhibition by selegiline could influence cardiac effects of moderate obesity. Our results show that moderate obesity improves cardiac contractility parameters. This effect might be mediated by altered Ca^2+^ homeostasis and increased TNF concentration. However, the beneficial effects of moderate obesity are attenuated by selective MAO-B inhibition by selegiline.

## 2 Results

### 2.1 HFD increased body weight, did not affect heart rate or blood pressure, however, improves parameters related to cardiac contractility and mechanoenergetics; whereas selegiline treatment normalized contractility and mechanoenergetics affected by HFD

The effect of HFD and selegiline treatment on body weight, food and caloric intake were described in our previous paper (Bou-Teen et al., 2021). The body weight was significantly elevated in HFD and HFD + S groups from week 16. At the end of the six months of feeding period body weight of control rats was 574 ± 17 g, control + S rates was 602 ± 18 g, HFD rats were 656 ± 34 g (+14.4 %), while HFD + S rats were 632 ± 16 g (+10.2 %). Data shows moderate obesity in HFD groups. Food intake was lower in HFD and HFD + S groups resulting in simi-lar caloric intake in all groups. Chronic selegiline treatment had no influence on body weight or food intake (Bou-Teen et al., 2021). Heart weight, heart rate, left ventricular and arterial systolic and diastolic pressures were not affected by HFD. Selegiline decreased arterial systolic and diastolic pressure and left ventricular end-systolic pressure regardless of the HFD treatment (Table 1).

**Table 1:** Basic in-vivo parameters. Hemodynamic parameters were measured at week 25 of the treatment. CON: control diet; CON + S: control diet and selegiline treatment; HFD: High fat, high sucrose diet; HFD + S: high fat, high sucrose diet and selegiline treatment. ∗: p < 0.05 vs. CON.

	CON (n = 6)	CON + S (n = 6)	HFD (n = 6)	HFD + S (n = 6)
Body weight (g)	574 ± 17	602 ± 18	656 ± 34∗	632 ± 16∗
Heart weights/tibia length (mg/mm)	41.3 ± 1.7	40.4 ± 1.8	42.4 ± 1.7	43.4 ± 1.1
Heart rate (bpm)	408 ± 8	387 ± 13	412 ± 14	393 ± 13
Arterial systolic pressure (mmHg)	174 ± 2	146 ± 11∗	175 ± 8	159 ± 8∗
Arterial diastolic pressure (mmHg)	138 ± 2	119 ± 8∗	135 ± 6	130 ± 7∗
Left ventricular end-systolic pressure (mmHg)	164 ± 4	143 ± 10∗	166 ± 8	154 ± 9∗
Left ventricular end-diastolic pressure (mmHg)	9 ± 1	8 ± 1	9 ± 1	8 ± 1
End systolic pressure-volume relationship (ESPVR) (mmHg/μl)	2.4 ± 0.1	2.7 ± 0.1	3.4 ± 0.1∗	2.7 ± 0.2
Preload recruitable stroke work (PRSW) (mmHg)	95.1 ± 2.4	86.0 ± 5.7	118.4 ± 8.8∗	92.3 ± 5.4
dP/dtmax – EDV (mmHg/s/μl)	55.5 ± 2.3	52.8 ± 3.8	69.2 ± 6.6∗	50.9 ± 2.9
Stroke work (mmHgxμl)	15448 ± 361	11398 ± 796	16178 ± 979	12708 ± 1251
Mechanical efficiency (%)	68.5 ± 2.0	70.1 ± 1.2	75.1 ± 1.6∗	71.3 ± 2.1
dPdt max (mmHg/sec)	9941 ± 376	7550 ± 517	10622 ± 399	9138 ± 1158
dPdt min (mmHg/sec)	−11463 ± 470	−9675 ± 547	−12250 ± 524	−9675 ± 547
Cardiac Output (mL/min)	58 ± 3	52 ± 3	59 ± 4	52 ± 3

In our in vivo model, moderate obesity had no detrimental effect on the cardiac functional parameters. On contrary, HFD significantly increased load-independent cardiac contractility parameters, such as ESPVR, PRSW, and dPdt – EDV, as well as altered ventricular mechanoenergetics (increased mechanical efficiency). However, when selegiline was administered along with HFD, this effect was completely abolished as these parameters normalized to the control values.

As we previously described (Nagy et al., 2018), HFD increased the adipose tissue ratio, a change that selegiline was able to reverse (Fig. S15). HFD-fed animals exhibited a downward trend in adiposity following selegiline treatment; however, this decrease was not statistically significant and animals remained moderately obese.

Results of the study indicated that the administration of the HFD diet led to a substantial reduction in the percentages of both bone and muscle tissue. However, a comparison of total bone and muscle volume showed that this decrease did not represent a loss of bone and muscle mass due to selegiline treatment.

### 2.2 Selegiline did not affect mitochondrial ROS production or oxygen consumption in HFD in vivo but reduced effect of HiChol in vitro

**Fig. 1:**
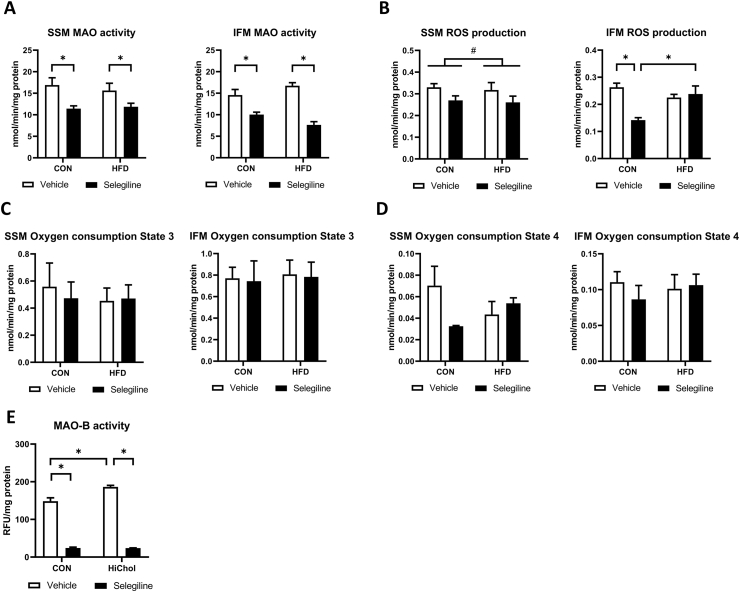
Selegiline reduces MAO-B activity in HFD diet, but it has no effect on mitochondrial ROS production or oxygen consumption. MAO-B activity (n = 12) of isolated subsarcolemmal (SSM) and interfibrillar (IFM) mitochondria from left ventricle samples (A). ROS production (n = 5) of isolated subsarcolemmal (SSM) and interfibrillar (IFM) mitochondria from left ventricle samples (B). Oxygen consumption in state 3 (OXPHOS) (n = 5) of isolated subsarcolemmal (SSM) and interfibrillar (IFM) mitochondria from left ventricle samples (C). Oxygen consumption in state 4 (in LEAK) (n = 5) (D) of isolated subsarcolemmal (SSM) and interfibrillar (IFM) mitochondria from left ventricle samples. MAO-B activity of H9c2 cells in vitro (Three independent experiment, n = 4) (E). Data are presented as mean ± SEM. (∗p < 0.05, #P < 0.05).

To investigate the effect of chronic HFD on mitochondrial redox functions, we measured MAOs activity, ROS production and oxygen consumption in cardiac SSM and IFM. Selegiline reduced cardiac MAO-B activity of both control and HFD rats, while neither selegiline nor HFD modulated cardiac mRNA expression of MAO isoforms (Fig. S2). Selegiline treatment significantly reduced the ROS production in the IFM and the oxygen consumption of the SSM of control diet-fed rats (Fig. 1A). We found that HFD did not in-crease mitochondrial and MAO-associated ROS production in SSM. In IFM selegiline de-creased ROS production in control but not in the HFD groups which means that selegiline-treatment was unable to reduce MAO-associated ROS in HFD compared to the control (Fig. 1B). HFD did not affect mitochondrial oxygen consumption in state 3 (Fig. 1C) and state 4 (Fig. 1D). Obesity did not result in increased cardiac nitrosative stress, however increased expression of catalase and reduced the antioxidant enzyme MnSOD (Fig. S3). Similar to adult rat cardiomyocytes, H9c2 cardiomyoblasts express mRNA (Fig. S4) of both MAO isoforms. To confirm the direct effect of HFD on MAO-B-related ROS production in the myocardium we treated H9c2 cells with a chemically defined hypercholesterolemic supplement (HiChol) which mimics cholesterol and fatty acid levels observed in rats with obesity (Makkos et al., 2019). HiChol treatment elevated MAO-B-related ROS production in H9c2 cell lysates, which was reduced by selegiline (Fig. 1E).

### 2.3 Selegiline may influence cardiac apoptosis in control chow-fed animals, with no effect on other cell death or survival mechanisms

**Fig. 2:**
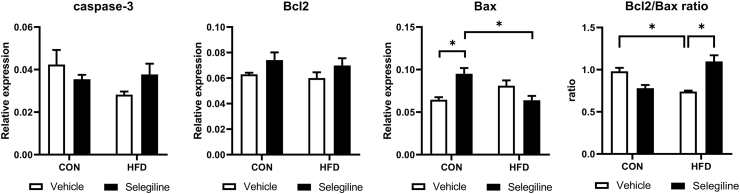
Effect of HFD and selegiline on the expression and phosphorylation status of apoptosis-related proteins. Western blot analysis of caspase-3 relative expression, normalized to GAPDH and Bcl2-to-Bax ratio. Necroptosis-related proteins: Western blot analysis of RIP1 and MLKL relative expression normalized to GAPDH and phosphor-RIP3/total-RIP3 ratio. Ferroptosis-related proteins: Western blot analysis of GPX4 normalized to GAPDH and phospho-HSP27/total-HSP27. Original Western blot imagery is in Figs. S8 and S13. Data are presented as mean ± SEM. (n = 3) (∗p < 0.05 vs. control; #p < 0.05 vs. HFD).

To assess the effect of HFD or MAO-B inhibition on programmed cell death and surviving mechanisms, we assessed the levels of main proteins of the canonical pathway of apoptosis, necroptosis, ferroptosis, autophagy and mitophagy. While caspase-3 activation was comparable among the experimental groups, Bcl-2/Bax ratio was decreased by HFD and was restored by selegiline. Interestingly, in control rats selegiline increased Bax expression which effect was not, however, seen in HFD animals (Fig. 2). These results suggest that selegiline may attenuate apoptotic sensitization in moderate obesity. Neither HFD nor selegiline influenced ferroptosis, necroptosis (Fig. S5), and autophagy (Fig. S6A), which execution may also be dependent on actual ROS levels in the cell (Dhalla et al., 2022; Ravingerova et al., 2020). As a result of moderate obesity, the expression of Beclin, a molecule involved in mitophagy, decreased significantly. This change was also detectable in our in vitro functional measurement, in the form of a reduction in mitophagy as a result of HiChol treatment (Fig. S6B–C).

### 2.4 Cardiac inflammation in HFD is not alleviated by selegiline

**Fig. 3:**
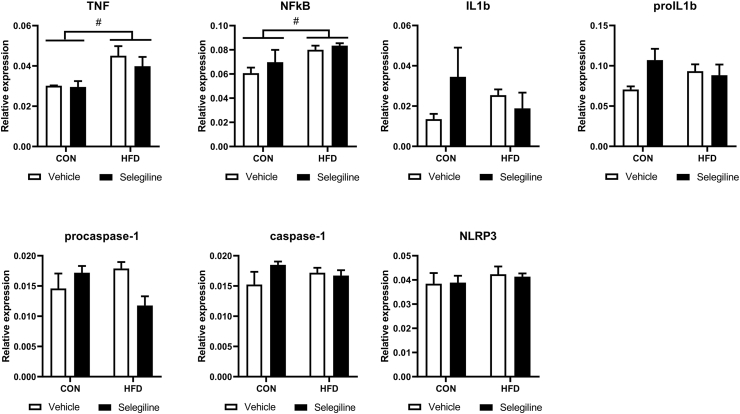
Effect of HFD and selegiline on the expression of inflammation-related proteins. Western blot analysis of TNF, NF-κB, IL-1β, pro-IL-1β, procaspase-1, caspase-1, and NLRP3 relative expression normalized to GAPDH. Original Western blot imagery is in Fig. S9. Data are presented as mean ± SEM. (n = 3) (∗p < 0.05 vs. control; #p < 0.05, two-way ANOVA, vs. control diet).

Since inflammation is an important pathophysiological aspect of CMD (Pereira and Alvarez-Leite, 2014; Yang et al., 2006; McMurray et al., 2016b; Bartekova et al., 2018; Geng et al., 2019a) we also assessed inflammatory parameters in the heart. HFD increased the inflammatory response as indicated by changes in the expression of the cleaved 17 kDa TNF fragment and NFkB p65, however, selegiline did not reduce these changes (Fig. 3, upper panels). No effect of HFD or selegiline treatment was observed on the expression of other markers of inflammation being related to pyroptotic cell death, such as pro-, or cleaved IL-1β (Fig. 3, upper panels), pro, and cleaved caspase 1, or NLRP3 (Fig. 3, lower panels) suggesting that HFD induces only a specific set of early inflammatory markers.

### 2.5 Calcium handling is disturbed in HFD

**Fig. 4:**
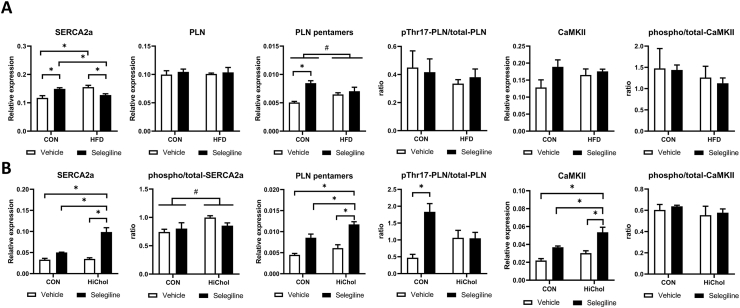
Effect of HFD or HiChol and selegiline on Ca^2+^ homeostasis. Expression of Ca^2+^ homeostasis-related proteins in vivo (A) and in vitro (B). Western blot analysis of phospho-PLN relative expression normalized to GAPDH, phospho-CaMKII/total-CaMKII ratio. Original Western blot imagery is in Figs. S10 and S11 Data are presented as mean ± SEM. (n = 3) (∗p < 0.05 vs. control; #p < 0.05, two-way ANOVA, vs. control diet).

CMDs affect cardiac function adversely, which is tightly connected to disturbances in intracellular Ca^2+^ homeostasis as a consequence of oxidative stress (Bou-Teen et al., 2021). Therefore, we assessed some of major proteins involved in calcium handling in the myocardium, from in-vivo samples and from rat H9c2 cardiac cells to confirm direct effect of moderate obesity and MAO-B inhibition on cardiac myocytes. Both HFD and selegiline modulated SER-CA2a expression in vivo, meanwhile its upstream modulators CaMKIIẟ, PLN and its phosphorylated forms were unaffected by HFD and selegiline. On the other hand, HFD initiated PLN pentamerization, indicating its direct interaction with the SERCA2a and thereby affecting its Ca^2+^ pumping function (Stokes et al., 2006) (Fig. 4A).

We also investigated gene expression of another important regulator of SERCA2a, sarcolipin (Fig. S16). Selegiline with high-fat diet may have increased sarcolipin expression, although statistical significance was not reached.

In H9c2 cells, expression of SERCA2a, CaMKIIẟ and PLN pentamerization were increased by selegiline and HiChol treatment. The phosphorylation of SERCA2a was promoted by HiChol, while PLN phosphorylation was elevated by selegiline in the control, but not in the HiChol group; and phosphorylation of CaMKIIẟ was not changed by HiChol or selegiline (Fig. 4B). These results suggest that cardiac Ca^2+^ homeostasis may be modulated by HFD and MAO-B inhibition. Therefore, we investigated whether these molecular changes alter cytosolic Ca^2+^ cycling of HL-1 cardiomyoblasts. Our results show that despite their effect on SER-CA2a and its modulators neither HiChol nor selegiline affected Ca^2+^ cycling in cardiomyoblasts (Fig. S7).

## 3 Discussion

This is the first study demonstrating a rather comprehensive evaluation of molecular events present in moderate obesity along with the effect(s) of MAO-B inhibition on cardiac function. Although our data seemingly include some controversial findings, such data might be important from the clinical point of view. The present findings indicate that HFD treatment resulted in moderate obesity in animals, which was associated with alterations in the percentages of fat, bone, and muscle tissue. Our study showed that moderate obesity did not affect major cardiac parameters, however, contractility-related parameters evidenced improved function in HFD-fed rats. Surprisingly, instead of synergistically im-proving cardiac function by reducing ROS production, MAO-B inhibition by selegiline completely abolished the beneficial effect of HFD. The Selegiline Summary of Product Characteristics (SMPC) (Electronic Medicines Compendium) lists a number of side effects, including bradycardia, arrhythmias, palpitations, angina pectoris and supraventricular tachycardia. These side effects are unlikely to be related to adipose tissue or the systemic effects of obesity. Therefore, they are not sufficient to explain the obesity paradox-reducing effect of selegiline. HFD as well as selegiline modulated the expression of SERCA2a, and its major regulator, PLN, although, calcium cycling was not affected by HC or selegiline treatment in cultured cardiac myocytes. Obesity was accompanied by a low-grade inflammation in the heart which was not reduced by selegiline. HFD also affected apoptotic processes, that were reversed by selegiline treatment. Autophagy, mitophagy, necroptosis and pyroptosis were not affected by either HFD or selegiline. In summary, even moderate obesity can influence the inflammatory processes at certain level as well as Ca^2+^ homeostasis of the heart, enhancing the positive effects of moderate obesity. MAO-B inhibition reduced this effect and therefore might not be of significant therapeutic value to alleviate cardiac consequences of CMDs.

As reported previously, selegiline did not reduce obesity, but reduced abdominal adiposity (Nagy et al., 2018), suggesting its beneficial metabolic effects. Therefore, we investigated how moderate obesity affects the heart, and if selegiline improves cardiac function in di-et-induced obesity. Contrary to our expectations, HFD did not have a negative effect on major cardiac parameters, suggesting that moderate obesity does not cause severe changes in cardiac physiology as opposed to models of morbid obesity (Haslam and James, 2005). Therefore, this model of moderate obesity resembles the healthy but obese human patients. In addition, alterations in cardiac parameters related to contractility were observed in our model of moderate obesity. Similar findings were reported previously, which were attributed to the increased expression of muscle contractile proteins by Adebayo et al. (2022). This is the first demonstration that MAO-B inhibition reverses functional changes induced by moderate obesity, that might be an adaptation to the altered loading conditions. The background of this phenomenon needs further investigation, as decreased ROS production by MAO inhibition, which we also observed in our model, has been reported to improve cardiac contractility (Wang et al., 2022). Therefore, functional benefit of MAO-inhibition might depend on the severity of obesity. It is important to note that we did not examine the right ventricular function or pulmonary circulation parameters in our studies. The effect of selegiline on these parameters is not well understood and therefore its investigation, either in a similar experimental setting or in a heart failure model, could be of interest.

Cardiac functions are tightly regulated by cardiac Ca^2+^ homeostasis (Bou-Teen et al., 2021), which is known to be disturbed by metabolic diseases (Carvajal et al., 2014). Effect of moderate obesity and MAO-B inhibition on cardiac Ca^2+^ homeostasis has not been investigated before. In our animal model of moderate obesity, HFD altered the expression of SERCA2a and affected its regulators PLN and Sln that could potentially impact cardiac function in a complex way. In our in vivo and in vitro models of obesity, selegiline influenced expression or activation of several key modulators of cardiac Ca^2+^ cycling. This suggests that MAO-inhibition has direct impact on cardiac Ca^2+^ handling of cardiac myocytes that might be independent of its obesity-limiting effect. We observed that simulated hypercholesterolemia does not change time parameters of Ca^2+^ cycling in cultured cardiomyocytes despite the molecular changes in Ca^2+^ cycling machinery, which suggests that either the in vitro system does not model metabolically healthy obesity well, or that the in vivo observed changes in cardiac function might be due to systemic effects. The latter hypothesis is supported by previous findings, that adipokines released from excessive metabolically healthy adipose tissue influence the change in Ca^2+^ cycling (Sawicka et al., 2016). Molecular effects of moderate obesity- and MAO-B inhibition on cardiac Ca^2+^ homeostasis need to be further investigated.

Here we found early signs of inflammation in the heart due to moderate obesity. Previously we also reported adipose inflammation in a similar in vivo model of obesity (Nagy et al., 2018), therefore, it is plausible that a low-grade systemic inflammation is present in this model of moderate obesity, which is in agreement with the current knowledge (Bartekova et al., 2018). Cardiac inflammation plays an important role in the development of obesity-induced cardiac derangements (Geng et al., 2019b). However, there is evidence suggesting that the low levels of inflammation observed in the obesity paradox, particularly TNF released from adipose tissue, is responsible for the improved cardiac function (Sawicka et al., 2016).

Hence, our data suggest that increased inflammation might underlie improved cardiac function in moderate obesity. However, selegiline reversed the improved function without affecting TNF levels, suggesting that selegiline may act downstream of TNF on the underlying molecular mechanism. Sawicka et al. highlighted that cardiomyocytes express TNFR2, through which TNF exerts cytoprotective effects, protecting the heart and promoting cardioprotective effects (Sawicka et al., 2016). Nevertheless, that the cardioprotective effect of this mild degree of inflammation needs verification.

Moderate obesity was previously shown to increase apoptosis (Li et al., 2005). In accordance, here we show that cardiac apoptosis induced by moderate obesity may have been reversed by selegiline treatment.

Our findings indicate that selegiline does not improve or, in some cases, may even impair the investigated cardiovascular and molecular parameters. It is plausible that the impact of selegiline on adipose tissue may contribute to the observed reduction in obesity-related complications, as adipose tissue has been identified as a key factor in the development of the obesity paradox.

A potential limitation of the study is the relatively low sample size in some in vitro experiments. Increasing group sizes may further confirm molecular background of cardiac effects of selegiline in obesity. A limitation of this study is that the lipid accumulation in the hearts was not investigated. Another limitation of this study is that higher than expected blood pressures were recorded in certain control animals, the reason of which has not been uncovered. However, the blood pressure measurement method or an unexpected interaction between pentobarbital anesthesia and altered lipid metabolism may have influenced the readings.

Findings of this study are notable from several clinical perspectives. Firstly, we report that obesity may not necessarily be associated with deteriorated cardiac function. Furthermore, we indicate a clinical situation where there may be consequences of pharmacological treatment of Parkinson's disease with MAO-B inhibitors in patients with obesity: selegiline may aggravate cardiometabolic diseases and thus increase the patient's susceptibility to cardiac damage. However, the translational value of this article could first be determined by conducting a longitudinal clinical study involving patients with Parkinson's disease.

In conclusion, our results showed that moderate obesity does not impair, and to some extent improves, cardiac function in HFD-fed rats. Surprisingly, selective MAO-B inhibitor selegiline blunts HFD-induced improvements in cardiac function. Both HFD and selegiline modulates Ca^2+^ homeostasis, and HFD enhances some markers of inflammation in the heart, and it is plausible that these processes may underlie the observed cardiac phenotype. HFD increased apoptosis, which was attenuated by selegiline. Oxidative stress, necroptosis, ferroptosis, and autophagy were not induced in the heart in our in vivo model of moderate obesity, and these alterations were not affected by MAO-B inhibition by selegiline.

In summary, selegiline has an adverse effect on the obesity paradox observed in moderate obesity, but further research is needed to fully elucidate the exact molecular mechanisms.

## 4 Materials and methods

### 4.1 Animal welfare

All animal care and experimental procedures conformed to Directive 2010/63/EU and were authorized by the regional animal health authority in Hungary (registration numbers: XIV-I-001/450-6/2012). Animal studies are reported in compliance with the ARRIVE guidelines (Kilkenny et al., 2010; McGrath and Lilley, 2015).

### 4.2 Diet-induced experimental obesity: study design

**Fig. 5:**
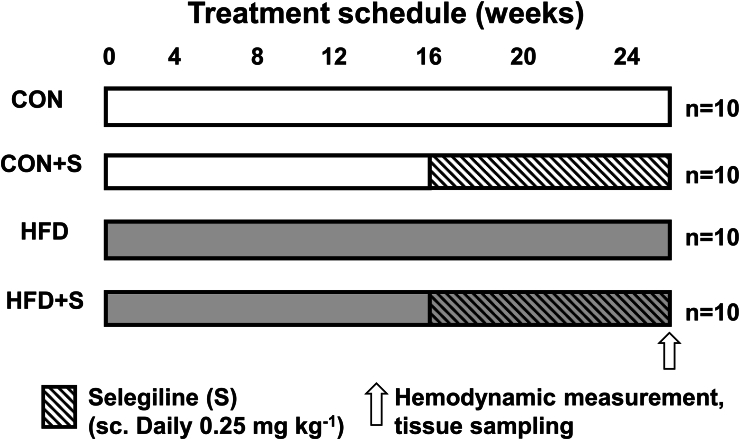
In vivo treatment schemes. Male Long-Evans rats were fed with CON (n = 10) diet or with HFD (n = 10) diet for 25 weeks. CON + S (n = 10) and HFD + S (n = 10) groups were treated with 0.25 mg x kg-1 selegiline daily (S) from week 16–24. Tissue sampling was performed after terminal process.

The details of the study design, diet of the animals and tissue samples were described previously (Nagy et al., 2018). After hemodynamic measurements, animals were sacrificed. Tissue samples were collected and stored at −80 °C (Fig. 5).

### 4.3 Blood pressure analysis

Arterial and left ventricular (LV) pressure analysis was performed according to a previously described protocol (Ruppert et al., 2020). With the use of a dedicated hemodynamic analysis program (PVAN; Millar Instruments), the following parameters were determined: systolic arterial blood pressure, diastolic arterial blood pressure, heart rate, LV end-systolic pressure, LV end-diastolic pressure.

### 4.4 Isolation of rat cardiac mitochondria

Subsarcolemmal (SSM) and interfibrillar (IFM) mitochondrial fraction were isolated as described previously (Koncsos et al., 2018; Palmer et al., 1977).

### 4.5 Mitochondrial oxygen consumption

Mitochondrial oxygen consumption was measured by high-resolution respirometry using an Oroboros Oxygraph-2k (Oroboros Instruments, Innsbruck, Austria) (Doerrier et al., 2018). Data were recorded by DatLab 4 software (Oroboros Instruments). Oxygen consumption of isolated mitochondria (0.05 mg/mL) were detected with glutamate (5 mM) & malate (5 mM) as respiratory substrates in the absence of ADP (state 4 or LEAK respiration) and presence of ADP (2 mM; state 3 or OXPHOS). The measurements were carried out in the following medium: 125 mM KCl, 20 mM HEPES, 2 mM K_2_HPO_4_, 1 mM MgCl_2_ and 0.025 % BSA (Bovine Serum Albumin; fatty acid-free).

### 4.6 Mitochondrial hydrogen peroxide production

Mitochondrial hydrogen peroxide (H_2_O_2_) production was measured by Amplex Ultra Red and horseradish peroxidase assay (Makrecka-Kuka et al., 2015; Tretter and Ambrus, 2014). The fluorescence was detected at 37 °C using a Photon Technology International (PTI; Lawrenceville, NJ, USA) Deltascan fluorescence spectrophotometer. The fluorescence signal was calibrated by 0.1 μM H_2_O_2_ at the end of each experiment. Glutamate (5 mM) & malate (5 mM) were added to the mitochondria (0.05 mg/mL) to measure H_2_O_2_ production in the absence and presence of ADP. The medium contained as follows: 125 mM KCl, 20 mM HEPES, 2 mM K_2_HPO_4_, 1 mM MgCl_2_ and 0.025 % BSA (Bovine Serum Albumin; fatty acid-free).

### 4.7 In vitro study design

In the present in vitro study, we used H9c2 rat cardiomyoblast adherent culture. The following groups were investigated: control (CON) (normocholesterolemic control; cell culture medium), vehicle (vehicle control; cell culture medium supplemented with the vehicle of HiChol supplementation), HiChol (cell culture medium supplemented with appropriate amount of Refeed® hypercholesterolemic medium containing 10 μM cholesterol (Makkos et al., 2019)), Selegiline (cell culture medium supplemented with 5 μg/mL selegiline), or HiChol + Selegiline (as above).

### 4.8 Cell culture

H9c2 rat cardiomyoblast cells were obtained from ECACC and cultured Dulbecco's modified Eagle's medium (DMEM) supplemented with 10 % heat-inactivated FBS (Invitrogen Life Technologies, Carlsbad, CA, USA), 2 mM glutamine, and 100 U/mL of penicillin and streptomycin. HL-1 mouse cardiomyoblast cells were obtained from Merck (Lot: RD1601001; SCC065) and cultured Claycomb Medium (Sigma, Rockwille, MD, USA) supplemented with 10 % HL-1 Cell Screened FBS (Sigma, Rockwille, MD, USA), 2 mM glutamine, 100 U/mL of penicillin and streptomycin, and 32 μg/mL norepinephrine solution. Cells were maintained at 37 °C in a humidified CO_2_ incubator and subcultivated according to the Producer's protocol. For simulated hypercholesterolemia, cells were seeded according to the experimental conditions and 24 h later, cells were refed with growth medium supplemented with Refeed® hypercholesterolemic medium supplement or its vehicle (0.3 % ethanol) with or without selegiline for another 48 h.

### 4.9 Detection of monoamine-oxidoreductase activity from isolated mitochondria

The measurement of MAO activity is based on the formation of its product H_2_O_2_ which is detected with Amplex UltraRed assay as described above. The MAO activity of isolated mitochondria was measured at 37 °C using Photon Technology International (PTI) Deltascan fluorescence spectrophotometer. Tyramine (500 μM) was added to the mitochondria as a MAO-substrate which initiated H_2_O_2_ production.

### 4.10 Detection of monoamine-oxidoreductase activity from cell homogenates

For the determination of MAO activities H9c2 cells were kept at 37 °C in a humidified CO_2_ incubator (5 % CO_2_). Cells were seeded at 10,000 cells/cm2 density and treated according to the in vitro study design. In the assay, MAO-A and MAO-B react with p-tyramine forming H_2_O_2_ which is determined by a fluorimetric method. H9c2 were pelleted and homogenized in Amplex UltraRed Measurement buffer by sonication (3 times 10 s on ice). Then, the cells were centrifuged at 1000 g for 10 min at 4 °C. The test was performed according to the manufacturer’ instructions. Fluorescent intensity was detected by multimode reader; excitation wavelength: 530 nm, emission wavelength: 585 nm.

### 4.11 Calcium cycling measurements

The Ca^2+^ cycles of mouse cardiomyoblast cells (HL-1) were quantified using the Fluo8 AM reagent. A detailed methodology is provided in the Supplementary materials.

### 4.12 Western blot

Protein expression in cardiac tissue and cell homogenates was assessed by Western blot as described earlier (Nagy et al., 2018; Lichý et al., 2020). Used antibodies are detailed in Table S1.

#### 4.12.1 Body composition analysis

In the phase of the analysis, segmentation of muscle and bone tissues was performed using whole-body CT reconstructions. The imaging data were acquired using a nanoScan SPECT/CT system (Mediso, Hungary), operating at 55 kV tube voltage, 500 ms exposure time, in helical mode, with an isotropic voxel size of 0.24 mm.

Image processing and segmentation were carried out in VivoQuant software (Invicro, USA), utilizing the 3D ROI tool. For both bone and muscle segmentation, the Connected Threshold algorithm was applied. The threshold range for bone tissue was set between 1500 and the maximum HU value, which allowed for fully automated segmentation with high accuracy. In contrast, muscle tissue was segmented using a threshold range of 600–900 HU. Due to the inclusion of adjacent organs within this range, extensive manual correction was necessary using the eraser tool to refine the muscle ROIs.

Following segmentation, the volumes of the identified tissues were calculated. These values were then normalized to body weight to derive the final quantitative results.

### 4.13 Statistical analysis

Statistical analysis was performed by two-way ANOVA using Tukey test as post hoc test only if F achieved P < 0.05, and there was no significant variance inhomogeneity by Prism 8.0.1 (GraphPad, GraphPad Software, USA).

## Author contributions

All authors contributed to the Conceptualization, Investigation, Formal analysis and Interpretation of data, or preparation of the manuscript, and gave final approval. Writing – Original Draft Preparation: Szabolcs Hambalkó, Csilla Pelyhe, Gábor B. Brenner and Zoltán Giricz. Zoltán Giricz. and Péter Ferdinandy agree to be accountable for all aspects of work ensuring integrity and accuracy.

## Ethics approval

All animal care and experimental procedures conformed to Directive 2010/63/EU and were authorized by the regional animal health authority in Hungary (registration numbers: XIV-I-001/450-6/2012).

## CRediT author statement

***Szabolcs Hambalkó*:** Writing - Original Draft, Investigation, Formal Analysis, Methodology, Visualization, Writing - Review & Editing **Csilla Pelyhe:** Writing - Original Draft, Investigation, Formal Analysis, Methodology, Visualization **Csenger Kovácsházi: Investigation, Methodology** **Bence Kenyeres:** Formal Analysis **Bernadett Kiss:** Investigation **Bence Ágg:** Formal Analysis **Tamás G. Gergely:** Fromal Analysis **Bennet Y. Weber:** Formal Anaysis **András Makkos:** Investigation ***Gábor* B. Brenner::** Writing - Original Draft **Tímea Komlódi:** Investigation, Methodology, Visualization **László Tretter:** Investigation, Methodology, Visualization **Csaba Horváth:** Investigation, Methodology, Visualization **Izabela Jarabicová:** Investigation, Methodology, Visualization **Adriana Adameová:** Investigation, Methodology, Visualization **Anikó Görbe:** Conceptualization, Methodology, Investigation **Paola Poggi:** Resources **Alexandros Chatgilialoglu:** Resources **Ildikó Horváth:** Formal Analysis **Domonkos Máthé:** Formal Analysis **Krisztián Szigeti:** Formal analysis **Attila Oláh:** Investigation, Methodology, Formal analysis **Tamás Radovits::** Investigation, Methodology, Formal analysis **Zoltán V. Varga:** Conceptualization, Methodology, Investigation **Béla Merkely:** Conceptualization **Gary F. Baxter:** Conceptualization **Rainer Schulz:** Conceptualization **Péter Ferdinandy:** Conceptualization **Zoltán Giricz::** Writing - Original Draft, Investigation, Formal Analysis, Methodology, Visualization. Writing - Review & Editing, Supervision.

## Funding

This research was funded by the 10.13039/501100018818National Research, Development and Innovation Office of Hungary (NKFIH; VEKOP-2.3.2-16-2016-00002, VEKOP-2.3.3-15-2017-00016, TÉT_16-1-2016-0057, National Heart Program NVKP, Grant/Award Number: NVKP_16-1-2016-0017, Project no. RRF-2.3.1-21-2022-00003 has been implemented with the support provided by the European Union National Laboratory Program Project no. RRF-2.3.1-21-2022-00003, 2020-1.1.5-GYORSÍTÓSÁV-2021-00011), 2020–1.1.6-JÖVŐ-2021-00013, OTKA-K-109737 and OTKA-K-107803 to PF K139237 to AG, K139105 to ZG and OTKA-FK-134751 to ZVV and OTKA-K-134939 to TR. The research was also financed by the EU COST Action BM1203 EU-ROS. Project no. RRF-2.3.1-21-2022-00003 has been implemented with the support provided by the 10.13039/501100000780European Union. TKP2021-EGA-23 has been implemented with the support provided by the Ministry of Innovation and Technology of Hungary from the National Research, Development and Innovation Fund, financed under the TKP2021-EGA funding scheme. This research was funded by the Research Excellence Programme of the National Research, Development and Innovation Office of the Ministry of Innovation and Technology in Hungary (TKP/ITM/NKFIH). The research was financed by the Thematic Excellence Programme (2020–4.1.1.-TKP2020) of the Ministry for Innovation and Technology in Hungary, within the framework of the Therapeutic Development and Bioimaging thematic programmes of the Semmelweis University. Bennet Y Weber was supported by the 250 years of Excellence Semmelweis scholarship (EFOP-3.6.3-VEKOP-16-2017-00009). Bennet Y. Weber was supported by the 2024-2.1.1-EKÖP-2024-00004 University Research Scholarship Programme of the Ministry for Culture and Innovation from the source of the National Research, Development and Innovation Fund.

The work was supported by the European Union's Horizon 2020 research and innovation programme under grant agreement No 739593. Z.G., Z.V.V. and É.S were supported by a János Bolyai Research Scholarship of the 10.13039/501100003825Hungarian Academy of Sciences and by the ÚNKP-19-4 and ÚNKP-20-4-II-SE-20; and B.Á. was supported by the ÚNKP-20-4-I-SE-7; New National Excellence Program of the 10.13039/501100015498Ministry for Innovation and Technology. A.A. was a receipt of 09I03-03-V04-00231/European Union - Next generation EU. RS was supported by Deutsche Forschungsgemeinschaft (DFG, German Research Foundation) [Project number 268555672 – SFB 1213, Project B05]. R.S. was funded by Deutsche Forschungsgemeinschaft (DFG, German Research Foundation), project number 268555672—SFB 1213, project B05. BK was supported by THE DOCTORAL STUDENT SCHOLARSHIP PROGRAM OF THE CO-OPERATIVE DOCTORAL PROGRAM. AM was supported by ÚNKP-23-4-II-SE-34; New National Excellence Program of the 10.13039/501100015498Ministry for Innovation and Technology.

## Declaration of competing interest

The authors declare the following financial interests/personal relationships which may be considered as potential competing interests: Peter Ferdinandy reports financial support was provided by National Research, Development and Innovation Office of Hungary. Andras Makkos reports financial support was provided by National Research, Development and Innovation Office of Hungary. Aniko Gorbe reports financial support was provided by National Research, Development and Innovation Office of Hungary. Zoltan Giricz reports financial support was provided by National Research, Development and Innovation Office of Hungary. Zoltan V Varga reports financial support was provided by National Research, Development and Innovation Office of Hungary. Tamas Radovits reports financial support was provided by National Research, Development and Innovation Office of Hungary. Bence Agg reports financial support was provided by National Research, Development and Innovation Office of Hungary. Bence Kenyeres reports financial support was provided by National Research, Development and Innovation Office of Hungary. Rainer Schulz reports financial support was provided by Deutsche Forschungsgemeinschaft. Peter Ferdinandy reports a relationship with Pharmahungary Group that includes: board membership. Zoltan Giricz reports a relationship with Pharmahungary Group that includes: employment. Gary F. Baxter reports a relationship with Pharmahungary Group that includes: consulting or advisory. Rainer Schulz reports a relationship with Amgen Inc that includes: speaking and lecture fees. Rainer Schulz reports a relationship with Recordati Pharma GmbH that includes: speaking and lecture fees. Rainer Schulz reports a relationship with Sanofi that includes: speaking and lecture fees. If there are other authors, they declare that they have no known competing financial interests or personal relationships that could have appeared to influence the work reported in this paper.

## Data Availability

The datasets generated during and/or analysed during the current study are available from the corresponding author on reasonable request.
