# Supplementary material for: MAO-B inhibition by selegiline blunts cardiac functions improved by high-fat diet: Role of inflammation, apoptosis, and calcium-handling
Source: Curr Res Pharmacol Drug Discov. 2025 Nov 11;9:100237. doi: 10.1016/j.crphar.2025.100237 (PMC12666686; doi:10.1016/j.crphar.2025.100237)
Supplement: Multimedia component 1 [file mmc1.pdf]

Supplementary figures

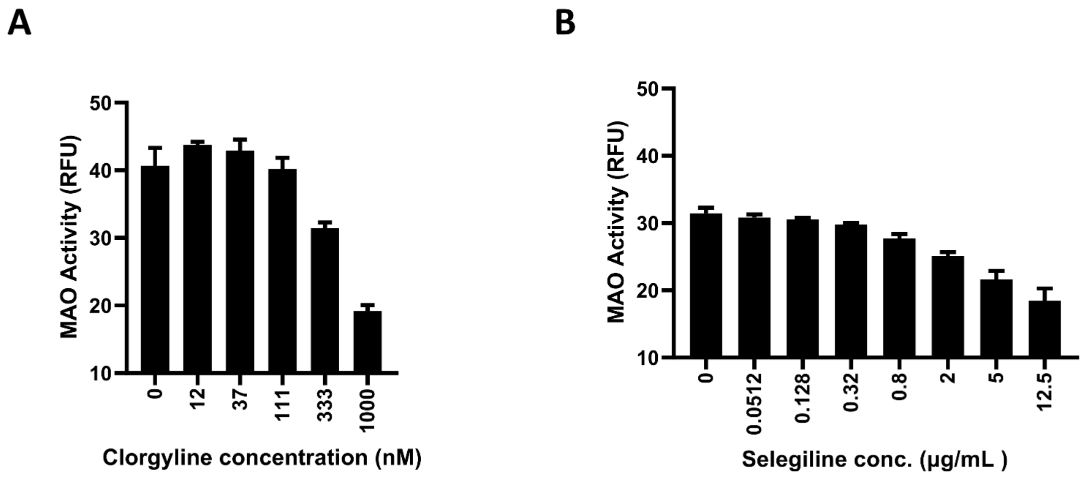

Figure S1. Optimization of MAO activity measurement. Clorgyline dose-response curve (A) for the determination of effective MAO-A inhibitor concentration. Selegiline dose-response curve (B) with the use of 333 nM clorgyline for the determination of effective selegiline concentration. (n=2) (* p<0.05 vs. control).

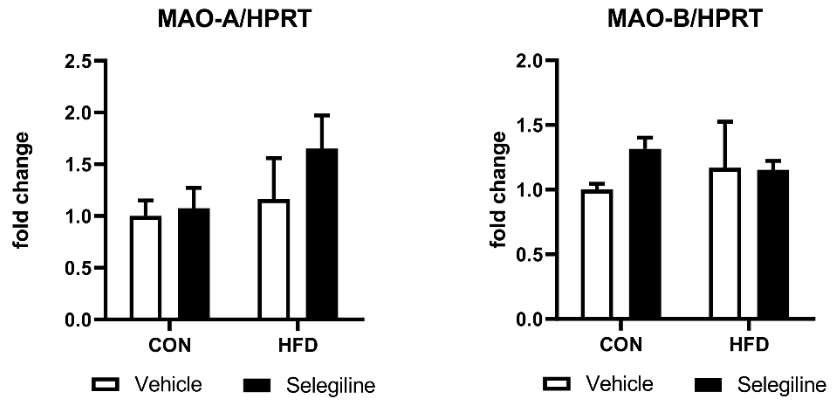

Figure S2. HFD or selegiline treatment did not change the mRNA expression of MAO isoforms. pPCR analysis of MAO-A and MAO-B relative gene expression normalized to HPRT. Data are presented as mean ± SEM. (n=4-5)

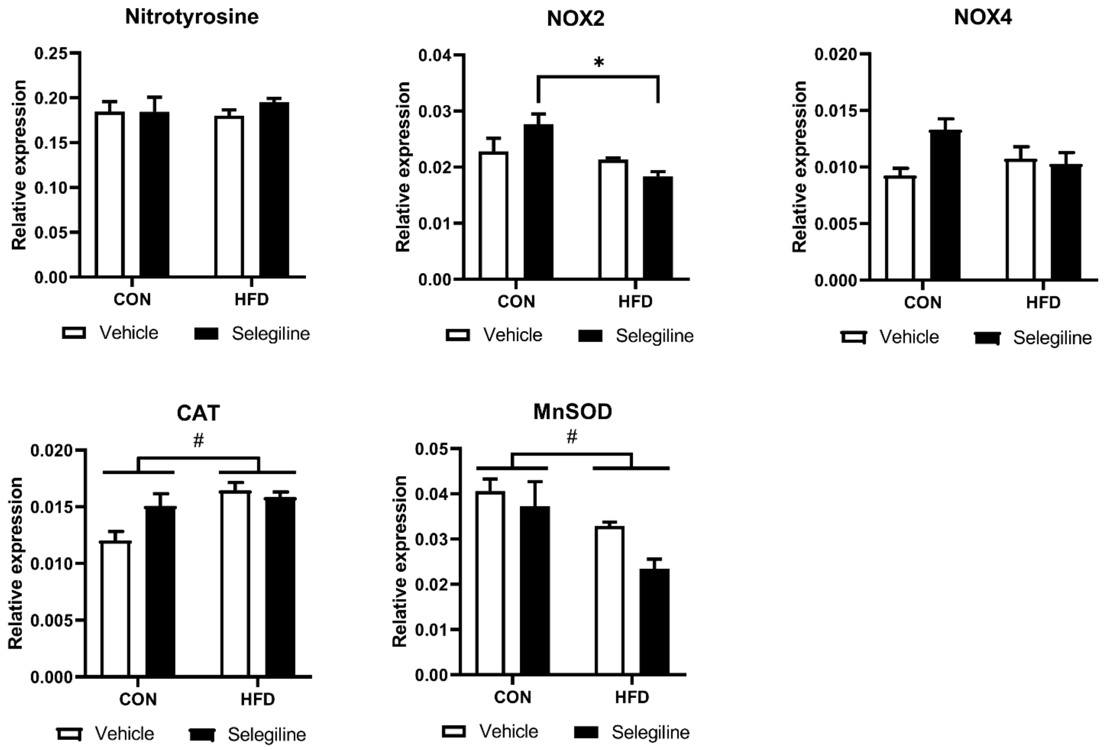

Figure S3. Effect of HFD diet and selegiline on nitro-oxidative stress and on the expression of antioxidant enzymes. Western blot analysis of NO_2Tyr, NOX2, NOX4, CAT, and MnSOD relative expression normalized to GAPDH. Data are presented as mean ± SEM. (n=3) (* $p<0.05$ vs. control; # $p<0.05$, two-way ANOVA, vs. control diet).

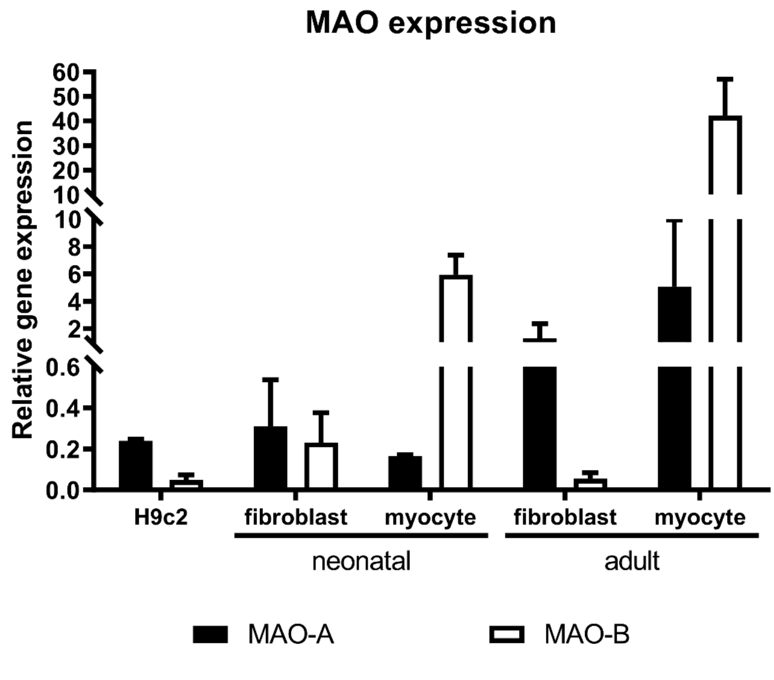

Figure S4. Relative mRNA expression of MAO-A and MAO-B in H9c2 cardiomyoblast cell lines and isolated cardiac cells of different ages. qPCR analysis of MAO-A and MAO-B relative gene expression normalized to HPRT in non-differentiated H9c2 cell line (H9c2), neonatal and adult rat cardiac fibroblasts, as well as in neonatal and adult rat cardiomyocytes. Data are presented as mean ± SEM. (n=2-3)

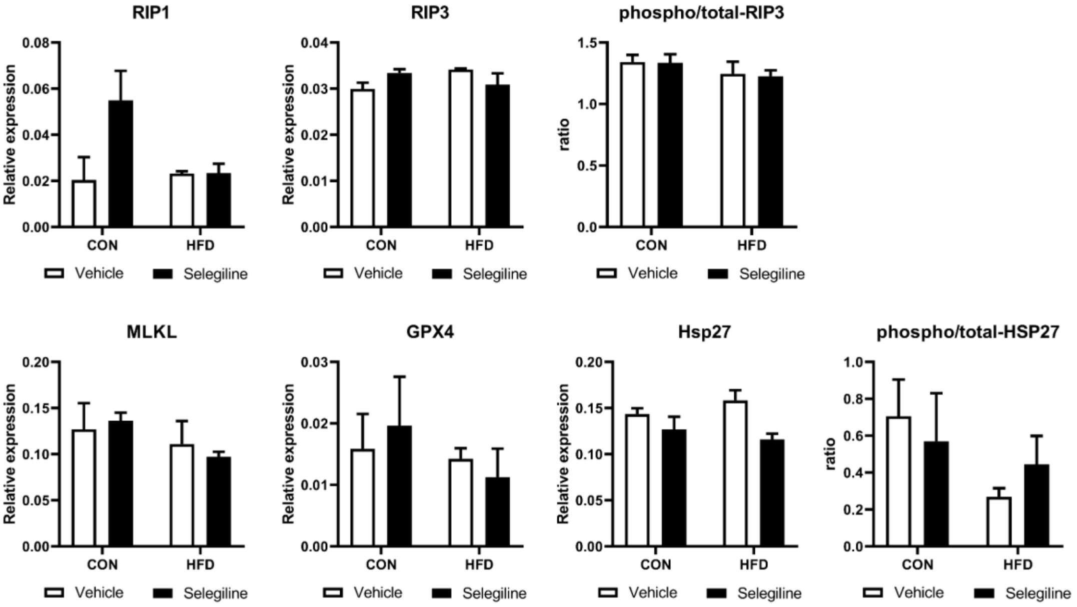

Figure S5. Effect of HFD and selegiline on the expression and phosphorylation status of necroptosis and ferroptosis-related proteins. Necroptosis- related proteins: western blot analysis of RIP1 and MLKL relative expression normalized to GAPDH and phosphor-RIP3/total-RIP3 ratio. Ferroptosis-related proteins: western blot analysis of GPX4 normalized to GAPDH and phospho-HSP27/total-HSP27. Data are presented as mean ± SEM. (n=3) (* $p<0.05$ vs. control; # $p<0.05$ vs. HFD).

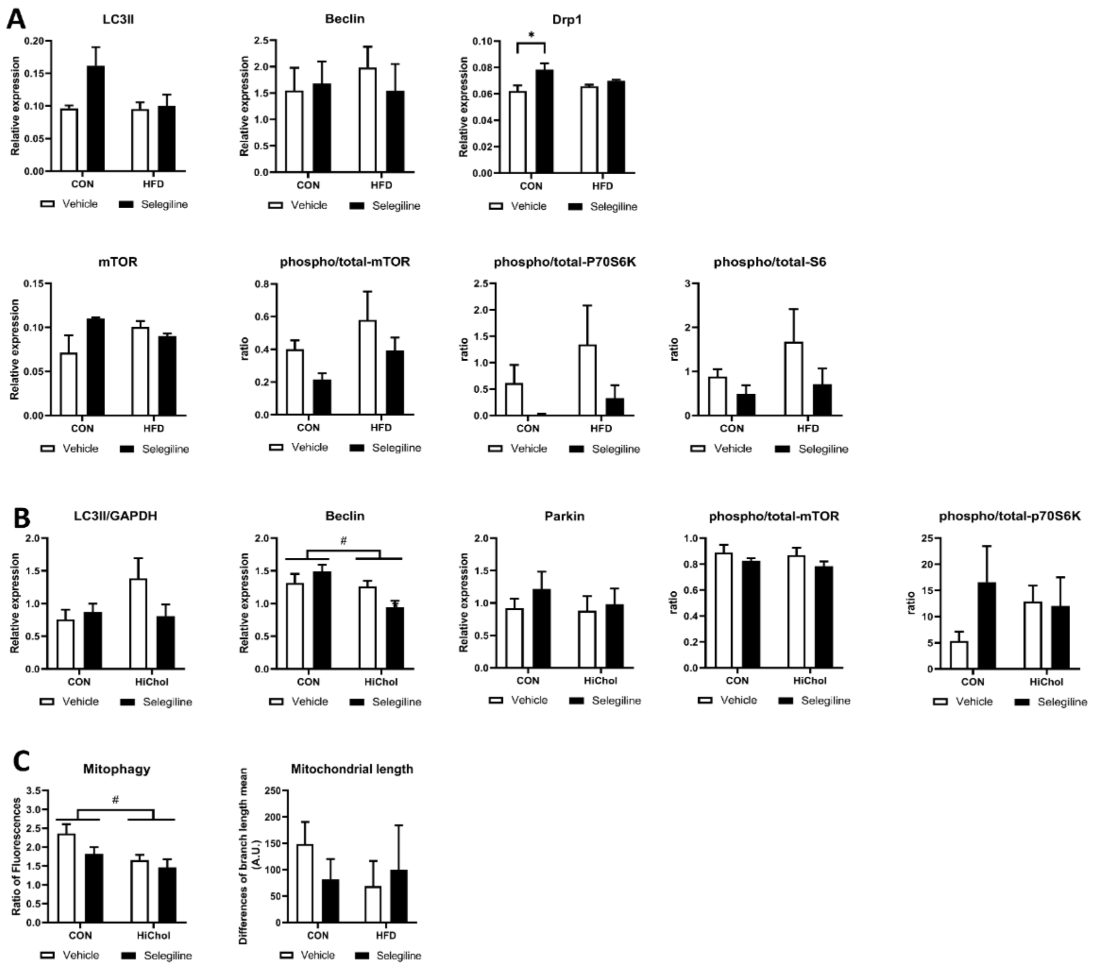

Figure S6. Effect of HFD and selegiline on the autophagy- and mitophagy. A) Effect of HFD and selegiline on the expression and phosphorylation status of autophagy- and mitophagy-related proteins. Western blot analysis of LC3II, Beclin, Drp1 relative expression normalized to GAPDH, p-mTOR/mTOR ratio, p-p70S6K/p70S6K ratio, and p-S6/S6 ratio. Data are presented as mean ± SEM. (n=3) (* p<0.05 vs. control). B) Effect of HiChol treatment and selegiline on the expression of autophagy and mitophagy-related proteins in H9c2 cells. Western blot analysis of LC3II, Beclin, and Parkin relative expression normalized to GAPDH. phosho-mTOR/mTOR, and phospho-p70S6K/p70S6K ratio. Data are presented as mean ± SEM. (n=5) (* p<0.05 vs. control; # p<0.05, two-way ANOVA, vs. control diet). C) Assessment of mitophagy and mitochondrial morphology. Results were calculated from the fluorescent intensities of 48 hours samples.

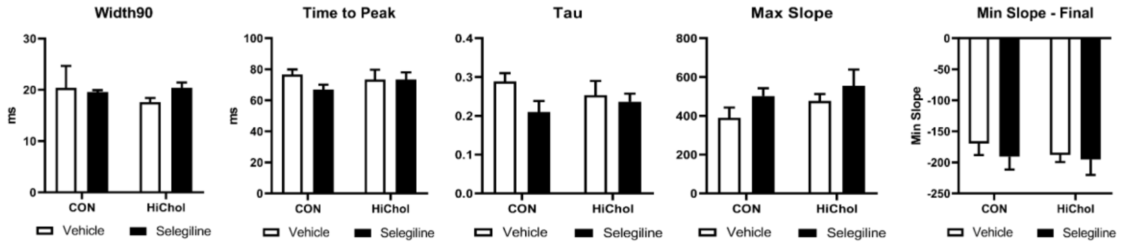

Figure S7. Effect of HiChol and selegiline on Ca2+ homeostasis in vitro. Confocal microscopy analysis of Ca2+ cycles of HL-1 mouse cardiomyoblasts. Data are presented as mean ± SEM. (n=6)

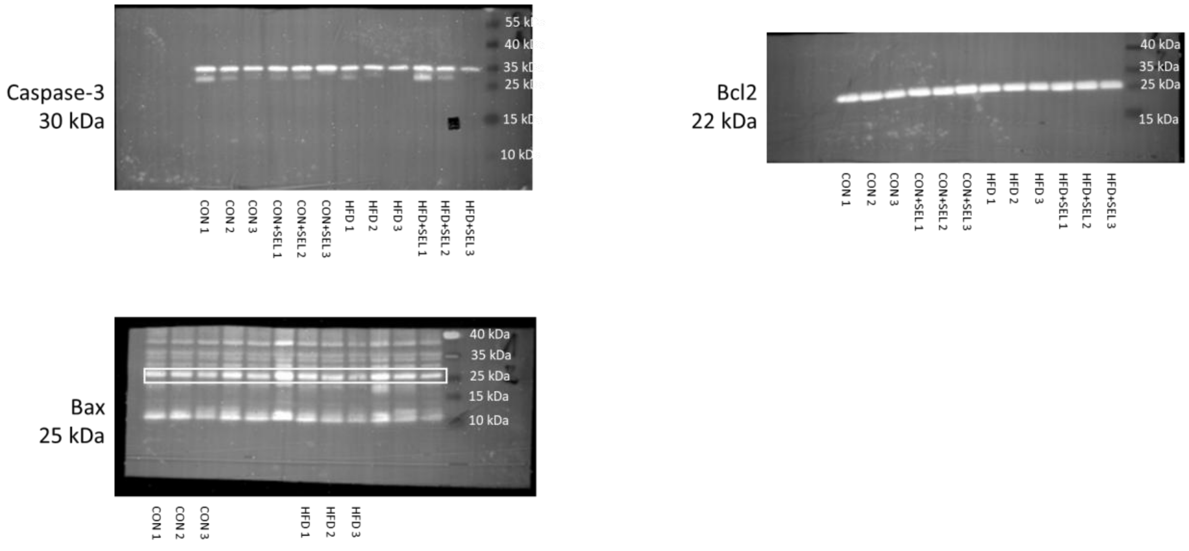

Figure S8: Whole blots of Figure 2.

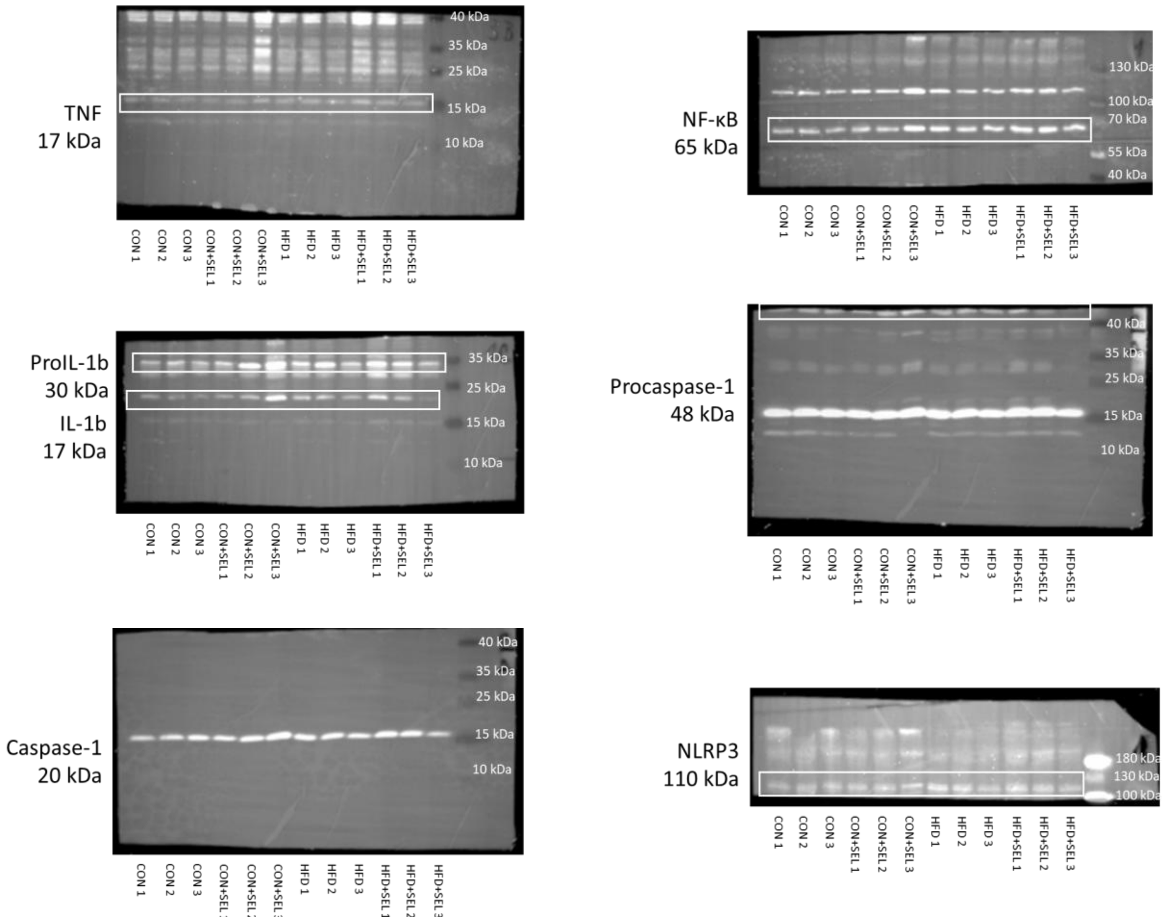

Figure S9: Whole blots of Figure 3.

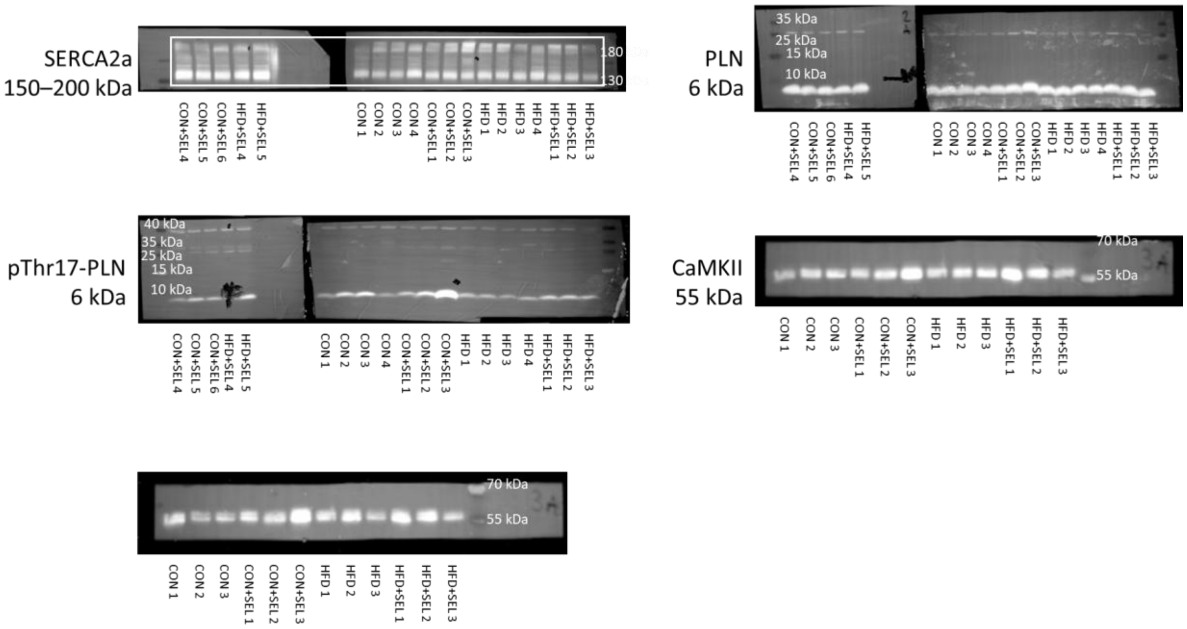

Figure S10: Whole blots of Figure 4A.

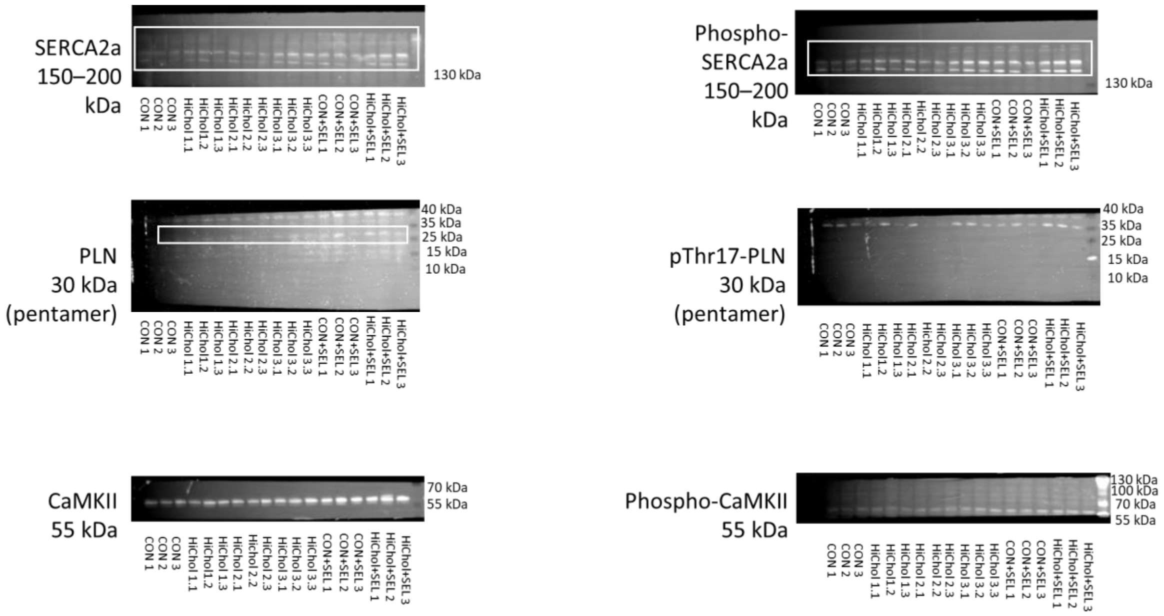

Figure S11: Whole blots of Figure 4B

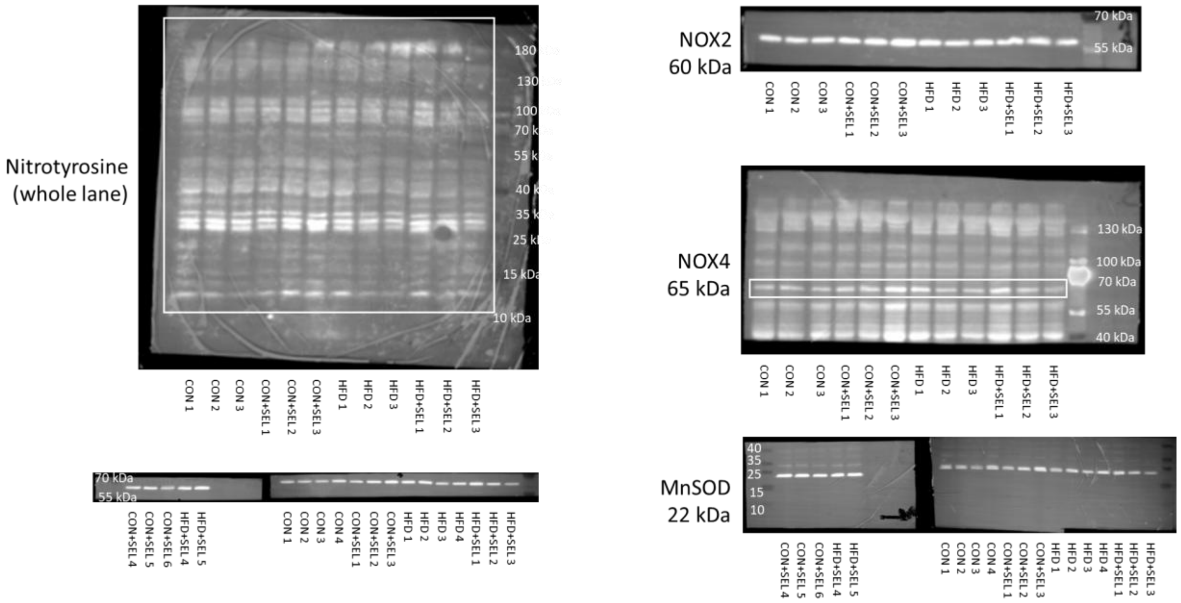

Figure S12: Whole blots of Figure S3.

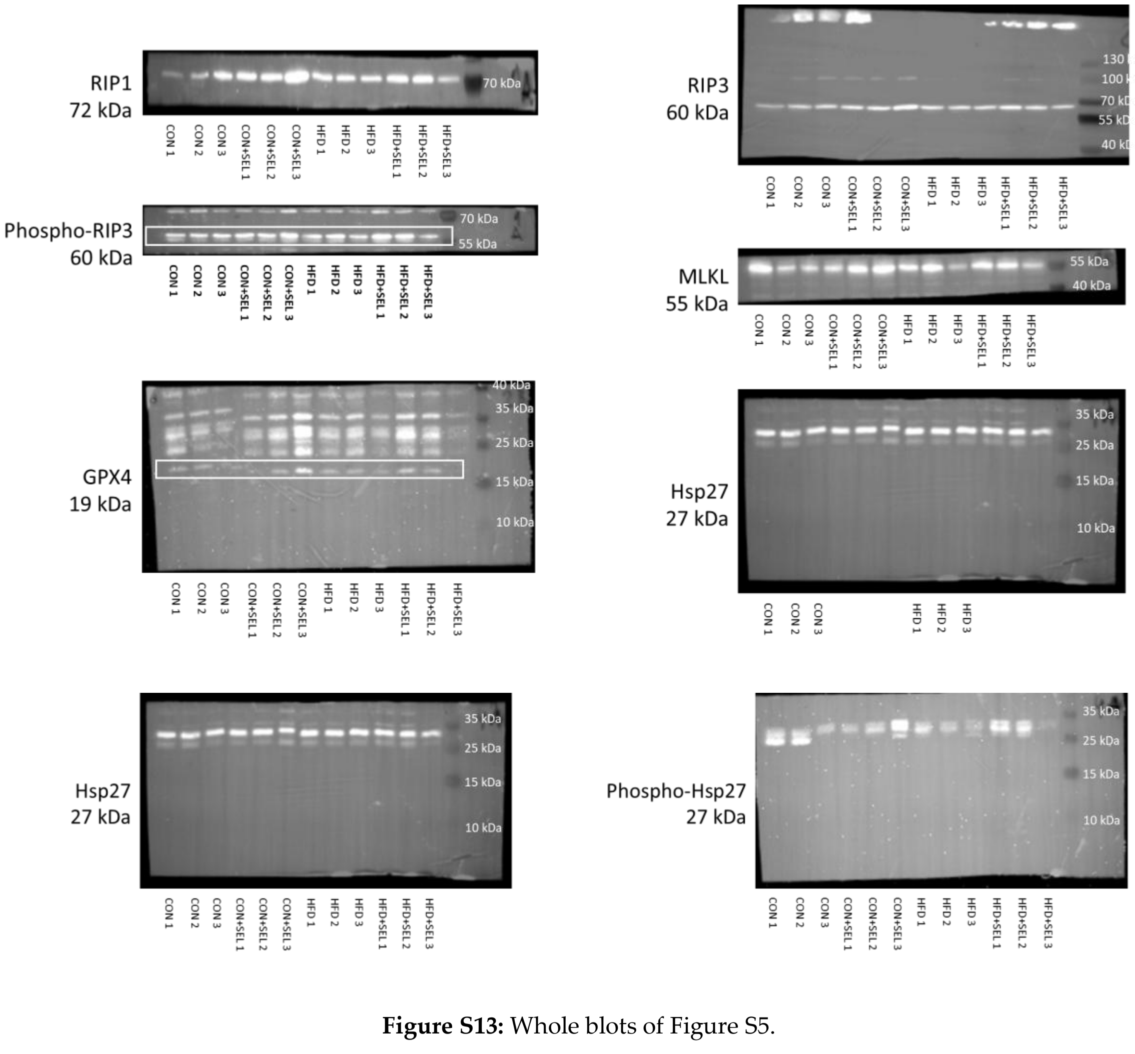

Figure S13: Whole blots of Figure S5.

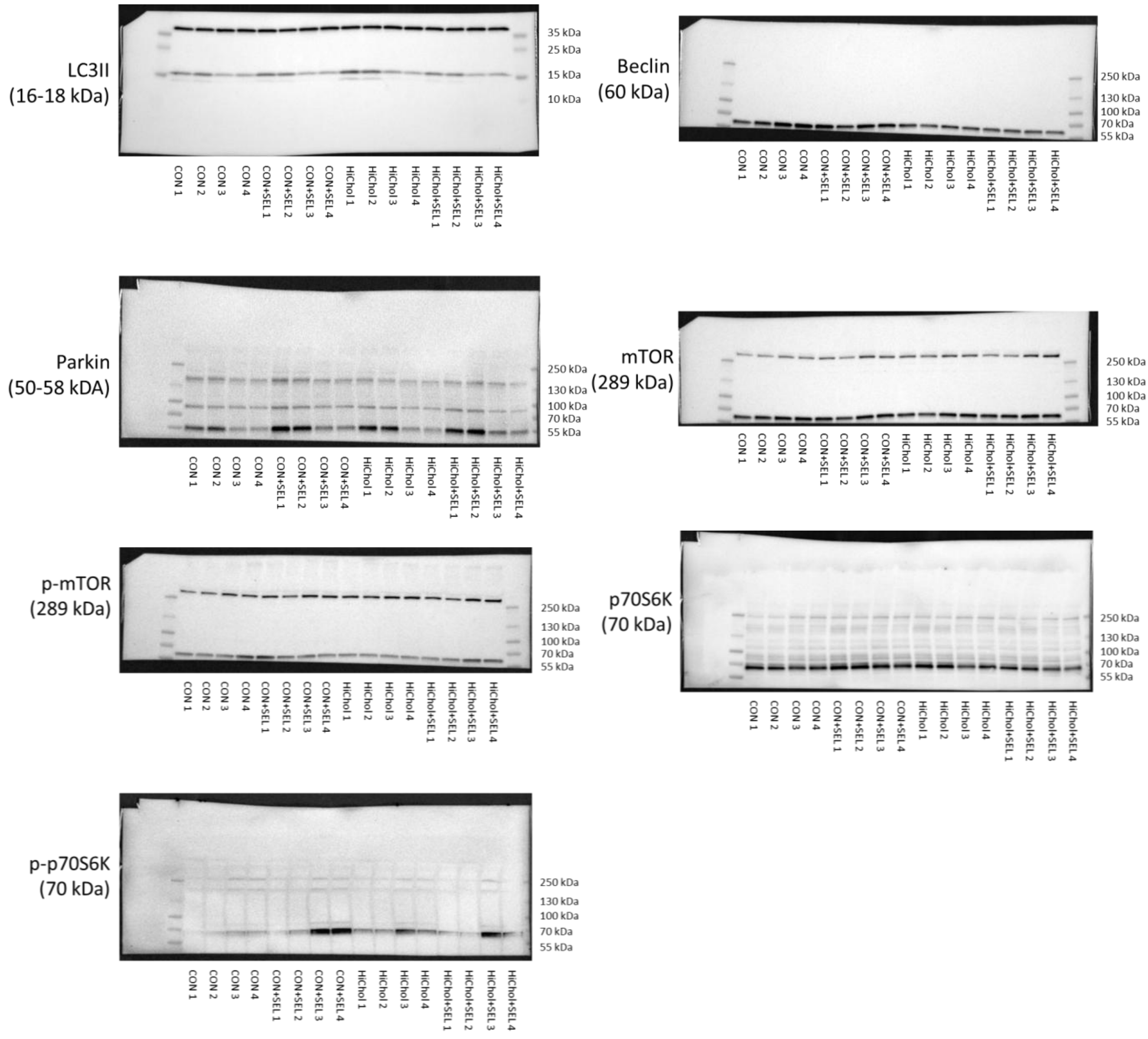

Figure S14: Whole blots of Figure S6.

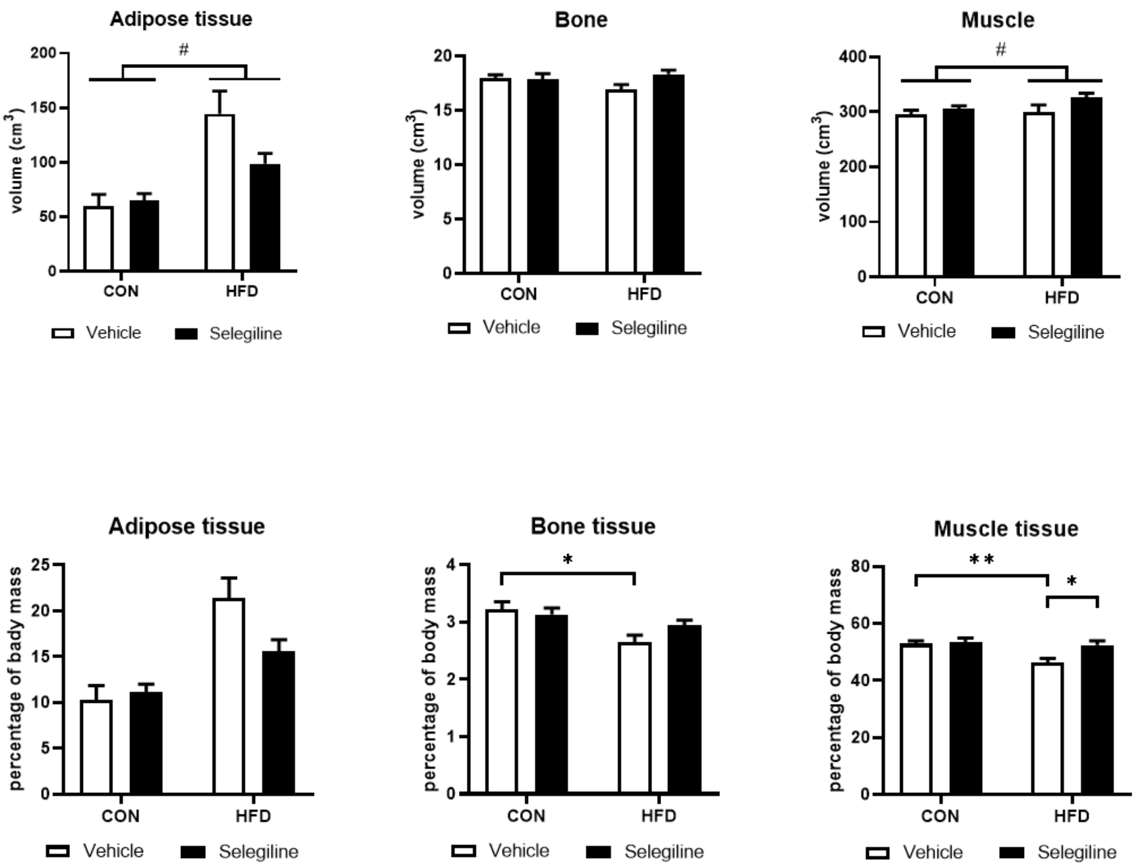

Figure S15: Results of the Body composition analysis. The top row shows the volume of adipose tissue, bone, and muscle, while the bottom row shows the percentage of adipose tissue, bone, and muscle to total body weight. Data are presented as mean ± SEM. (n=8) (* $p<0.05$ vs. control; # $p<0.05$, two-way ANOVA, vs. control diet).

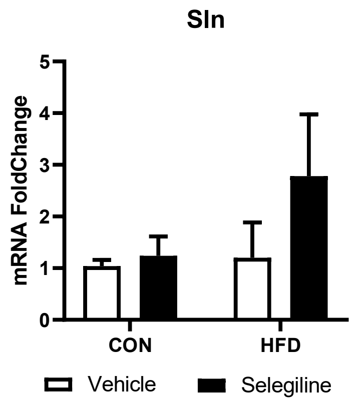

Figure S16. Effect of HFD diet and selegiline on gene expression of sarcolipin. qPCR analysis of sarcolipin relative expression normalized to HPRT. Data are presented as mean ± SEM. (n=4-5) (* $p<0.05$ vs. control; # $p<0.05$, two-way ANOVA, vs. control diet).

Supplementary Materials and Methods

Calcium cycling measurements

Mouse cardiomyoblast cells (HL-1) were cultured as described in Materials and Methods section. Before calcium measurements, cells were seeded for 24 hours onto 10-well Greiner CELLview cell culture slide (Greiner Bio-One, Germany). Cells were treated with hypercholesterolemic supplement and/or selegiline as described above. After treatment, HL-1 cells were loaded for 30 min at 37 °C with 5.0 μM Fluo-8 AM in normal Tyrode solution twice. Extracellular Fluo-8 AM was removed by washing the culture with normal Tyrode solution twice. Measurements were performed in normal Tyrode solution in a cell culture incubator (37 °C, 5% CO2, >85% humidity). Calcium signal measurements were carried out by following time-lapse sequences of cellular fluorescence recordings with Leica SP8 confocal microscope at approximately 100 frame/s. For Ca2+ imaging cells were excited with the 488 nm laser line (0.5% intensity) and emission was measured between 497 and 597 nm. During measurements cells were paced at a frequency of 1 Hz by 80 V/5 mm field stimulation for 1 min. The intensity profiles of paced cells were acquired by defining intracellular area as regions of interest (ROIs) of single cells and extracting the values for further analysis. Recordings were evaluated using LabChart 8 (v8.1.6) software using the PeakAnalysis module with a following setting: Analysis type: General, Preset: General, Minimum Peak: 3 S.D. Peaks with width or less than 100 ms and more than 1100 ms were excluded from the analysis. When more than 14 peaks had to be excluded from an evaluation, the whole recording was excluded.

Table S1. List of antibodies used.

**Antibody name**	**Catalog number**	**Manufacturer**
LC3	#4108	Cell Signaling Technology
mTOR	2972	Cell Signaling Technology
phospho-mTOR	2971	Cell Signaling Technology
total p70S6K	SAB2500736	Sigma
phospho-p70S6K	S6436	Sigma
Beclin	3495	Cell Signaling Technology
S6	2217	Cell Signaling Technology
phospho-S6	2211	Cell Signaling Technology
GAPDH	5174	Cell Signaling Technology
Bax	ab182734	Abcam
Bcl-2	SAB4500003	Sigma-Aldrich
Caspase-1	ab179515	Abcam
Caspase-3	#9662	Cell Signaling Technology
CaMKIIδ	ab181052	Abcam
pThr287-CaMKIIδ	ab171095	Abcam
CAT	#14097	Cell Signaling Technology
Drp-1	#8570	Cell Signaling Technology
GPX4	ab255789	Abcam
Hsp27	#2442	Cell Signaling Technology
pSer82-Hsp27	#2401	Cell Signaling Technology
IL-1β	ab9722	Abcam
MLKL	ab243142	Abcam
mTOR	#2983	Cell Signaling Technology
pSer2448-mTOR	#2971	Cell Signaling Technology
NFκB	#8242	Cell Signaling Technology
NLRP3	#15101	Cell Signaling Technology
NO2Tyr	#9691	Cell Signaling Technology
NOX2	ab129068	Abcam
NOX4	ab133303	Abcam
pThr17-PLN	SAB1306553	Sigma-Aldrich
p53	#32532	Cell Signaling Technology
PLN	A010-14	Badrilla
RIP1	#3493	Cell Signaling Technology
RIP3	#15828	Cell Signaling Technology
pSer229-RIP3	ab195117	Abcam
SERCA2a	#9580	Cell Signaling Technologies
pThr484-SERCA2a	#PA5-117240	Thermo Fisher Scientific
MnSOD	#13141	Cell Signaling Technology
TNF-α	ab66579	Abcam

List of abbrevations:

Acsl4	Acyl-CoA Synthetase Long Chain Family Member 4
Ankrd28	Ankyrin Repeat Domain 28
Atg5	Autophagy related protein 5
Bak1	BCL2 Antagonist/Killer 1
Bax	B-cell lymphoma 2-associated X protein
Bbc3	BCL2 Binding Component 3
Bcl-2	B-cell lymphoma 2
Bcl2l11	BCL2 Like 11
Becn1	Coiled-Coil Myosin-Like BCL2-Interacting Protein
CaMKIIδ	Calcium-calmodulin-dependent protein kinase II
CAT	catalase
cMyBPC	Myosin binding protein C
CON + S	Control + selegiline
CON	Control
Crebrf	CREB3 Regulatory Factor
Cu-ZnSOD	Copper- and zinc-containing superoxide dismutase
Dicer1	Dicer 1 ribonuclease III
DMEM	Dulbecco's modified Eagle's medium
Drp1	Dynamin-related Protein
Fam178a	SMC5-SMC6 complex localization factor 2
Foxp2	Forkhead box protein P2
GAPDH	Glyceraldehyde 3-phosphate dehydrogenase
GPX4	glutathione peroxidase 4
H2O2	Hydrogen peroxide
HFD + S	High-fat diet + selegiline
HFD	High-fat diet
HiChol	high cholesterol supplement
HPRT	Hypoxanthine-guanine phosphoribosyltransferase
HSP27	Heat shock protein 27
IFM	interfibrillar mitochondrial fraction
Il1a	Interleukin 1 alpha
IL-1β	Interleukin 1 beta
Jazf1	JAZF zinc finger 1
LC3	microtubule-associated proteins 1A/1B light chain 3B
LC3II	lipid modified form of microtubule-associated proteins 1A/1B light chain 3B
LV	Left ventricular
MAO	Monoamine oxidases
Map1lc3a	Microtubule-associated proteins 1A/1B light chain 3A
MLKL	Mixed Lineage Kinase Domain Like Pseudokinase
MnSOD	Manganese superoxide dismutase
mTOR	Mechanistic target of rapamycin
Nfe2l2	Nuclear Factor, Erythroid 2 Like 2
NFkB	Nuclear factor-kappa B
NLRP3	NOD-, LRR- and pyrin domain-containing protein 3
NO2Tyr	Nitrotyrosine

NOX2	NADPH oxidase 2
NOX4	NADPH oxidase 4
Nr3c1	Nuclear receptor subfamily 3, group C, member 1
Opa1	Mitochondrial Dynamin Like GTPase
p70S6K	Ribosomal protein S6 kinase beta-1
Pcdhac2	Protocadherin Alpha Subfamily C, 2
phospho-CaMKII	phosphorylated Calcium-calmodulin-dependent protein kinase II
phospho-PLN	phosphorylated phospholamban
p-mTOR	phosphorylated mechanistic target of rapamycin
p-p70S6K	phosphorylated Ribosomal protein S6 kinase beta-1
p-S6	phosphorylated Ribosomal protein s6
RIP1	Receptor-interacting serine/threonine-protein kinase 1
RIP3	The receptor-interacting protein kinase 3
ROS	reactive oxygen species
S6	Ribosomal protein s6
SERCA2a	sarcoplasmic/endoplasmic reticulum Ca^{2+} ATPase 2a
Sgk1	Serum/Glucocorticoid Regulated Kinase 1
Shc1	SHC Adaptor Protein 1
SQSTM1	sequestosome-1
SSM	Subsarcolemmal mitochondrial fraction
TNF	Tumor necrosis factor
Usp32	Ubiquitin Specific Peptidase 32
Ythdf3	YTH N(6)-methyladenosine RNA binding protein 3
